# Synthetic Peptide CK2.3 Enhances Bone Mineral Density in Senile Mice

**DOI:** 10.4172/2572-4916.1000190

**Published:** 2018-06-30

**Authors:** John Nguyen, Hilary Weidner, Lora M. Schell, Linda Sequeira, Ryan Kabrick, Saurabh Dharmadhikari, Harold Coombs, Randall L. Duncan, Liyun Wang, Anja Nohe

**Affiliations:** 1 Department of Biological Sciences, University of Delaware, Newark, DE 19716, USA; 2 Department of Mechanical Engineering, University of Delaware, Newark, DE 19716, USA; 3 The Jackson Laboratory, Bar Harbor, ME 04609, USA

**Keywords:** Casein kinase II, Bone morphogenetic protein, Osteoporosis, Osteoblastogenesis, Osteoclastogenesis

## Abstract

**Background:**

Osteoporosis is a silent disease caused by low bone mineral density that results in bone fractures in 1 out of 2 women and 1 in 4 men over the age of 50. Although several treatments for osteopenia and osteoporosis are available, they have severe side effects and new treatments are desperately needed. Current treatments usually target osteoclasts and inhibit their activity or differentiation. Treatments that decrease osteoclast differentiation and activity but enhance osteogenesis and osteoblast activity are not available. We recently developed a peptide, CK2.3, that induces bone formation and increases bone mineral density as demonstrated by injection over the calvaria of 6 to 9-day-old mice and tail vein injection of 8-week-old mice. CK2.3 also decreased osteoclast formation and activity. However, these studies raise questions: does CK2.3 induce similar results in old mice and if so, what is the effective CK2.3 concentration and, is the bone mineral density of vertebrae of the spinal column increased as well?

**Methods:**

CK2.3 was systematically injected into the tail vein of female 6-month old mice with various concentrations of CK2.3: 0.76 μg/kg, 2.3 μg/kg, or 6.9 μg/kg per mice. Mice were sacrificed one week, two weeks, and four weeks after the first injection. Their spines and femurs were collected and analyzed for bone formation.

**Results:**

Femur and lumbar spine analyses found increased bone mineral density (BMD) and mineral apposition rate, with greater stiffness observed in femoral samples four weeks after the first injection. Histochemistry showed that osteoclastogenesis was suppressed in CK2.3 treated senile mice.

**Conclusions:**

For the first time, this study showed the increase of lumbar spine BMD by CK2.3. Moreover, it showed that enhancement of femur BMD was accompanied by increased femur stiffness only at medium concentration of CK2.3 four weeks after the first injection indicating the maintenance of bone’s structural integrity by CK2.3.

## Background

Bone remodeling is a dynamic process and the balance between osteoclast and osteoblast activity is key to the normal bone remodeling. Changes in this balance may cause many bone diseases, including osteoporosis. Age-related or senile osteoporosis is the most common type of osteoporosis especially in the elderly. Bone fractures are often the secondary complication in patients with hip fractures posing a serious threat to the patients. The mortality rate ranges from 10% to 40% during the first year after the fracture [1]. Osteoporosis affects more than 10 million adults in the United States [2]. Treatment of osteoporosis-related fractures is costly [3]. Current treatments for osteoporosis include bisphosphonates or hormone therapy; however these treatments exhibit some serious side effects including jaw pain, esophageal cancer, and stroke [4,5]. Therefore, a new treatment for osteoporosis with less severe side effects is needed to improve the quality of life for patients.

Recombinant human bone morphogenetic protein 2 (rhBMP2) was approved by the FDA in 2002 to treat spinal fusion. Studies show that BMP2 enhances bone mineralization and increases bone mineral density of lumbar spines [6]. However, long term usage of BMP2 results in decreasing bone mass [7]. This negative effect of BMP2 on bone loss is supported by studies that show the effect of BMP2 in enhancing osteoclastogenesis [8–11]. Previously our lab has demonstrated that casein kinase 2 (CK2) acts as a regulatory protein of BMP2 signaling pathway through its interaction with BMP type I receptor (BMPRIa) [12,13]. Our studies have uncovered three potential CK2-interacting sites on BMPRIa: 475–479 aa (site 1), 324–328 aa (site 2), and 213–217 aa (site 3) [13]. Blocking the interaction of CK2 and BMPRIa at site 3 showed increased osteoblastogenesis and mineralization [14,15]. Moreover, the injection of CK2.3 of 2.3 μg/kg per mice into the tail vein of 8-week old mice showed increased bone mineral density and the mineral apposition rate [14]. Primary pre-osteoclasts isolated from spleens of these mice showed decreased osteoclast differentiation and activity [14]. These studies raise the question about what concentration of CK2.3 is necessary to induce osteogenesis and decrease osteoclastogenesis. Furthermore, will CK2.3 induce bone formation in older mice and if so, is the bone stiffness also increased? Additionally, does CK2.3 directly affect osteoclasts in the femur and decrease their number?

In this study we investigate the effects of three different concentrations (0.76 μg/kg, 2.3 μg/kg, and 6.9 μg/kg per mice) of CK2.3 in 6-month-old mice. Our results show that all three concentrations of CK2.3 increased trabecular bone mineral density and mineral apposition rate. Moreover, the bone mineral density increased in the lumbar spine of the mice. In addition, all three concentrations of CK2.3 showed a decreased number of osteoclasts one week and two weeks after the first CK2.3 injections. Interestingly, an increase in femur shaft stiffness was detected only with the 2.3 μg/kg per mouse (medium) concentration four weeks after the first CK2.3 injection. In addition, an increase in osteocalcin expression, an osteoblast marker, was also detected only with the 2.3 μg/kg concentration four weeks after the first CK2.3 injection. In conclusion, the results showed that not only CK2.3 increased BMD but it also maintained and enhanced bone’s structural integrity.

## Materials and Methods

### Mouse injections

Retired breeders of female C57BL/6J mice were obtained from the Jackson Laboratory (Bar Harbor, ME). At 6 months of age, female mice (n=6/group) were injected in the tail vein once a day for five consecutive days with CK2.3 at, 0.76 μg/kg (low), 2.3 μg/kg (medium), and 6.9 μg/ kg (high) per mouse, or 50μl of PBS as a vehicle control. The choice of low, medium, and high concentrations was based on FDA guidelines [16]. According to the guidelines, an accepted drug should have less than a twofold difference in minimum lethal dose and minimum effective dose values. Thus, the tested concentrations should be twofold less and twofold higher than the minimum effective dose value. However, in order to be ensure the safety of CK2.3 we used threefold less (low concentration) and threefold higher (high concentration) than the minimum effective dose (medium concentration). The choice of medium concentration was based on our previous publication where we found 2.3 μg/kg per mouse was the effective dose [14]. At one week, two weeks, and four weeks after the initial injection, mice were sacrificed, and femurs and spinal cords were isolated.

### Bone mineral density

Analysis of volumetric bone mineral density (BMD) of femurs was measured by peripheral quantitative computed tomography (pQCT) [17].

### Single photon absorptiometry (SPA) of mouse lumbar spine

Bone mineral density of the lumbar spine was measured by single photon absorptiometry (SPA), as the X-ray penetrates through the sample in a single photon ray and is reflected onto a detector [18]. The lumbar spine was X-rayed from the anterior side with a Nomad Pro Handheld X-ray System. The distance (20 cm) between the handheld X-ray and the lumbar spine remained constant after each measurement. Single view lateral dorsal radiographs were imaged of each lumbar spine on a ScanX Duo digital imaging system (Air Techniques, Melville, NY). Exposure time was 0.40 sec. The kilovoltage peak (kVp) was 60. The milliampere-second (mAs) was 2.5. The pixel intensity (PI) was calculated for each radiograph in ImageJ to estimate BMD.

### Calculation of BMD

Two PIs (pixel intensities) of the background regions of interest (ROI) were measured using the “Measure” function of ImageJ. The average was then subtracted from the PI of the bone intensity ROI in order to obtain the BMD. PI is a measurement of a grey level value on a scale of 0 (black) to 255 (white) and has been shown to correspond with bone density (mineralized) or BMD in several other studies [19–21].

### Three-point bending test

Femur stiffness was analyzed by a three-point bending test using RSA-G2 Solids Analyzer (TA Instruments, New Castle, DE). The midshaft of the femur was compressed in the anterior-posterior direction with a lower support span of 4.5 mm and a loading rate of 0.05 mm/s. The instrument was set to stop when bone was fractured, or the compressive force reached 10N. The stiffness was defined as the slope of the linear region of the displacement vs. force graph.

### Histology sample preparation

Left femurs were fixed in 10% neutral buffered formalin for 48h and decalcified in 14% EDTA for 3–4 weeks. Chemical end-points were tested with ammonium hydroxide/ammonium oxalate (1:1 v/v). Paraffin embedding and sectioning were performed by the Histochemistry and Tissue Processing Core at Nemours/Alfred I. duPont Hospital for Children (Wilmington, DE).

Right femurs were fixed using 10% neutral buffered formalin for 24–48 hours at 4°C and embedded in methylmethacrylate (MMA) as previously described [14]. Once the femurs were polymerized into blocks, they were sectioned using a Buehler Diamond Watering Blade (10.2cm x 0.3mm) and sanded down.

### Immunostaining

The paraffin embedded of left femur samples were immersed in two changes of 100% xylene to deparaffinize. Then, samples were rehydrated with washes of 100% ethanol, 96% ethanol, 70% ethanol for 5 min each, and deionized water for 30 seconds. Antigen retrieval was performed by incubation with testicular hyaluronidase at 37°C for 30 minutes. After antigen retrieval the samples were incubated with 3%BSA for one hour at room temperature. Then they were fluorescently labeled overnight at 4°C with a mixture of primary antibodes goat polyclonal IgG osteocalcin (Santa Cruz Biotechnology, Dallas, TX) and rabit polyclonal IgG alkaline phosphatase (Santa Cruz Biotechnology). This was followed by a mixture of secondary antibodies with Alexafluor 488 donkey anti-goat IgG (Life Technologies, Waltham, MA) and Alexafluor 594 chicken anti-rabbit for one hour at room temperature. All antibodies were diluted in a 3% BSA solution. Bisbenzimide (Sigma-Aldrich, St. Louis, MO) was used as a nuclear stain for ten minute incubation. The coverslips were mounted using Airvol, as previously described [22,23]. Images were taken on Zeiss Axiophot (Zeiss, Oberkochen, Germany) at 200× total magnification and analyzed in ImageJ.

### Image analysis

The images collected were analyzed in ImageJ through the “Analyze Particles” function. Briefly, images were converted to 8-bit, then the threshold was adjusted in order to create a black and white image to eliminate excess background fluorescent staining from the bone. Three regions of interest that corresponded to bone or marrow cavity area were selected and measured for “mean” pixel intensity of each bone or marrow cavity region per image. The image area was selected based upon bone or marrow cavity availability. Pixel intensity was shown to be equivalent to fluorescent staining intensity [24]; therefore calculating the “mean” pixel intensity would directly correspond to the intensity of the osteocalcin stain within either the bone or marrow cavity region. Once intensities were measured, mean pixel intensities of each respective region (bone or marrow cavity) were calculated and averaged per respective concentration (PBS, Low, Medium, and High). Secondary control “mean” averages were subtracted from the “mean” averages of the samples in order to obtain an overall value that could be compared across the different concentrations for the osteocalcin staining intensity of the bone or marrow cavity.

### Tartrate-resistant acid phosphatase (TRAP) assay

Sectioned samples of left femurs were deparaffinized in xylene using 2 changes for 10 minutes each. Sections were then rehydrated with washes of 100% ethanol, 96% ethanol, 70% ethanol for 5 min each, and deionized water for 30 seconds. Samples were incubated in pre-warmed (37°C) TRAP staining solution (110 mM sodium acetate anhydrous / 50 nM sodium L-tartrate dibasic dehydrate, pH 4.7–5.0, 0.27 mM naphthol AS-MX phosphate and 6.7 mM fast red violet LB salt) for 1h at 37°C. Then, samples were counterstained with 0.02% fast green for 2 minutes and dehydrated through deionized water, 70% ethanol, 96% ethanol, 100% ethanol, and 2 changes of xylene, for 5 seconds each.

Once samples were dried, images of the growth plate and trabecular regions were taken using Zeiss Axiophot microscope in bright field at 100× total magnification with Nikon Digital Sight DS-Fi1 camera (Nikon, Tokyo, Japan). Three to four images were taken to get the whole growth plate region. Each purple TRAP+ spot was designated as an osteoclast. All of the osteoclasts in each image were counted and summed up (overlapping osteoclasts were excluded) to get the total number of osteoclasts in the growth plate. A blind count was conducted. The reported result was the average of blind count and non-blind count.

### Mineral apposition rate (MAR) assay

New bone formation was labeled with 100 μl of Calcein green (12 mg/mL) two days apart as described previously [14]. MAR was determined as previously described [14].

### Statistical data analysis

All data presented were analyzed using single factor analysis of variance (ANOVA), followed by Tukey-Kramer post-hoc test. All experiments were repeated three or more times and normalized to control. Error bars represent standard error of the mean (SEM).

## Results

**Figure 1:**
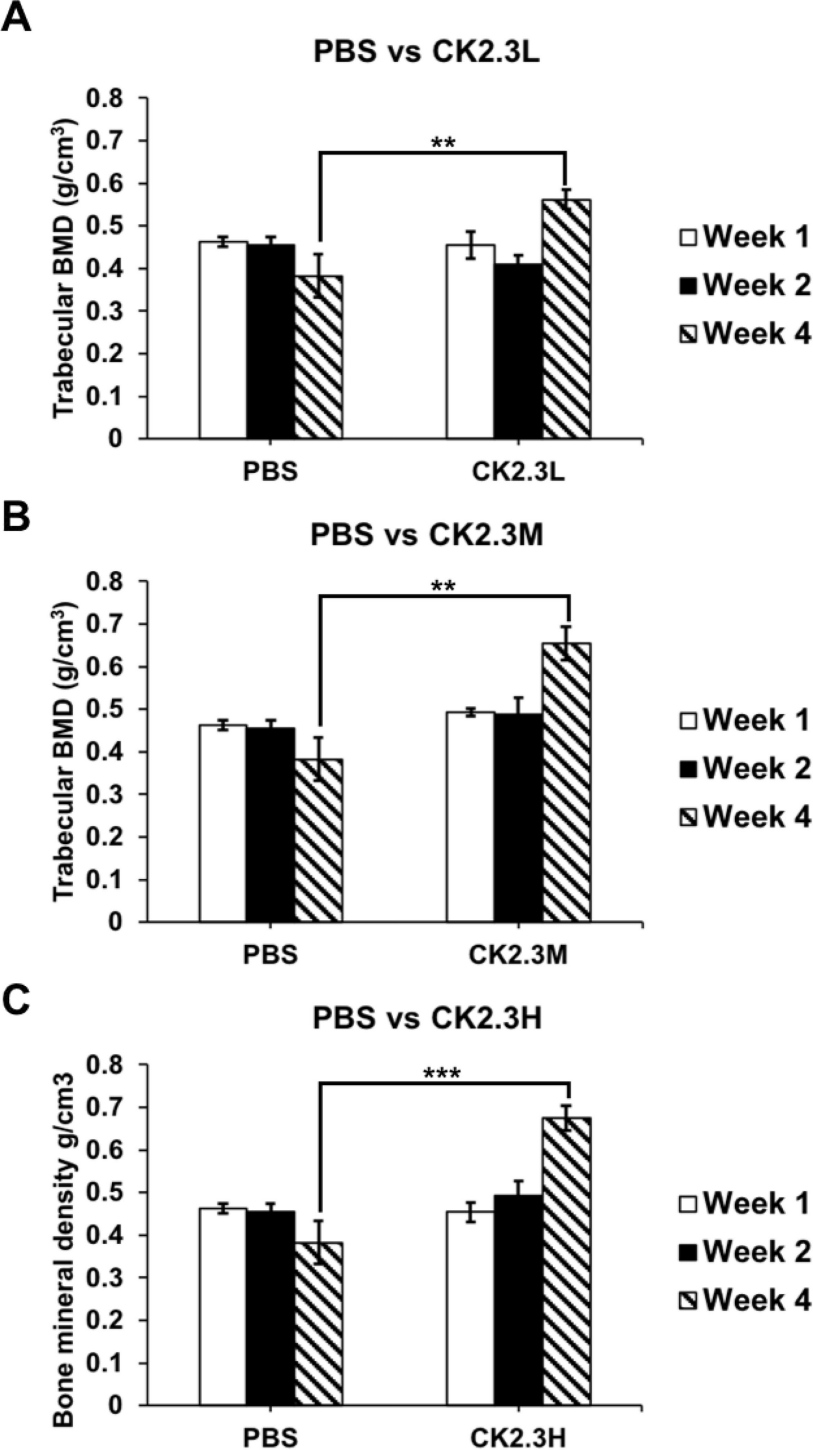
Injection of CK2.3 enhanced trabecular BMD four weeks after the first injection. CK2.3 didn’t affect trabecular BMD at earlier time points of one week and two weeks after the first injection at any concentration of injection. However, trabecular BMD increased four weeks after the initial injection at all concentrations of injection. Five to six mice were used per group of treatment at each time point. Statistically significance was performed by ANOVA followed by Tukey-Kramer (**and***=significant, p-value<0.01 and p-value<0.0001, respectively).

We report that CK2.3 increases trabecular BMD after 4 weeks of injection at a 2.3 μg/kg concentration [14]. We observed that CK2.3 significantly increased trabecular BMD four weeks after the initial injection at CK2.3L (n=6, 0.56 g/cm^3^ ± 0.022) (p-value<0.01), CK2.3M (n=6, 0.65 g/cm^3^ ± 0.040) (p-value<0.01), and CK2.3H (n=6, 0.67 g/ cm^3^ ± 0.028) (p-value<0.0001) when compared to PBS control injection (n=6, 0.38 g/cm^3^ ± 0.051) (Figure 1). BMD was similar between the PBS control (n=6, 0.46 g/cm^3^ ± 0.011 and 0.45 g/cm^3^ ± 0.018) and CK2.3injected group at CK2.3L (n=6, 0.46 g/cm3 ± 0.031 and 0.41 g/cm3 ± 0.023), CK2.3M ((n=6)0.49 g/cm3 ± 0.0087 and (n=5)0.49 g/cm^3^ ± 0.039), and CK2.3H (n=6, 0.45 g/cm^3^ ± 0.023 and 0.49 g/cm^3^ ± 0.034) one week and two weeks after the initial injection, respectively (Figure 1).

**Figure 2:**
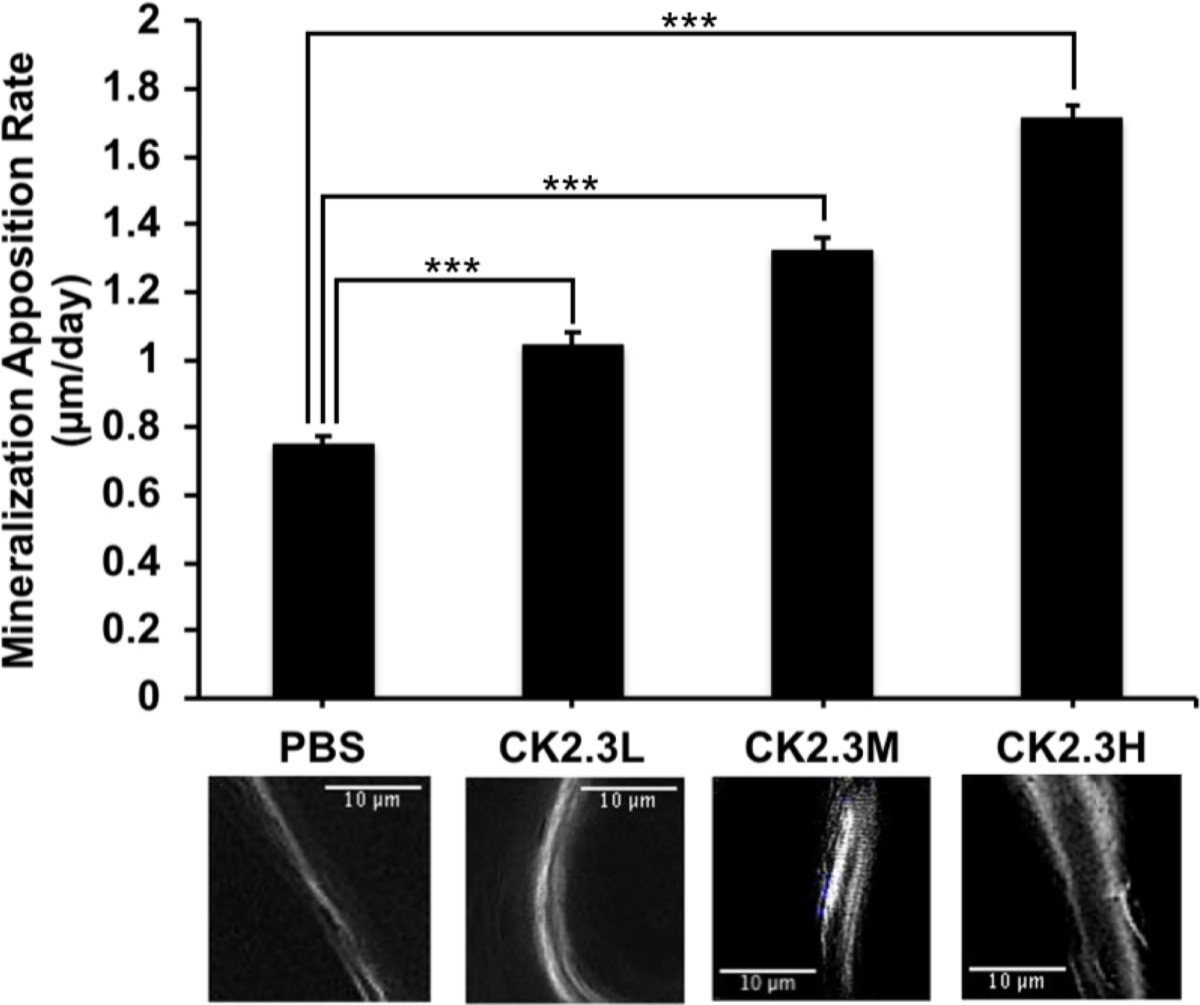
Injection of CK2.3 enhanced bone growth. MAR significantly increased in CK2.3 injected mice four weeks (p-value<0.0001) after the first injection. The graph represents the average bone growth in μm per day among the different concentrations. The images underneath the graph correspond with the above concentration, were taken at 100× total magnification and represent the trabecular bone region. Six mice were used per group of treatment. Statistically significance was performed by ANOVA followed by Tukey-Kramer (***=significant, p-value<0.0001).

Investigating bone formation, we observed that all CK2.3 doses had significantly increased MAR (p-value<0.0001) when compared to the PBS control (n=6, 7.75 μm/day ± 0.59) (Figure 2). It was observed that the increase of rate of bone formation was positively correlated with concentration of CK2.3. The CK2.3H (n=6, 19.17 μm/day ± 0.68) had the highest MAR and was significantly different from CK2.3M (n=6, 14.32 μm/day ± 0.78) that was also significantly different from CK2.3L (n=6, 11.71 μm/day ± 0.64).

**Figure 3:**
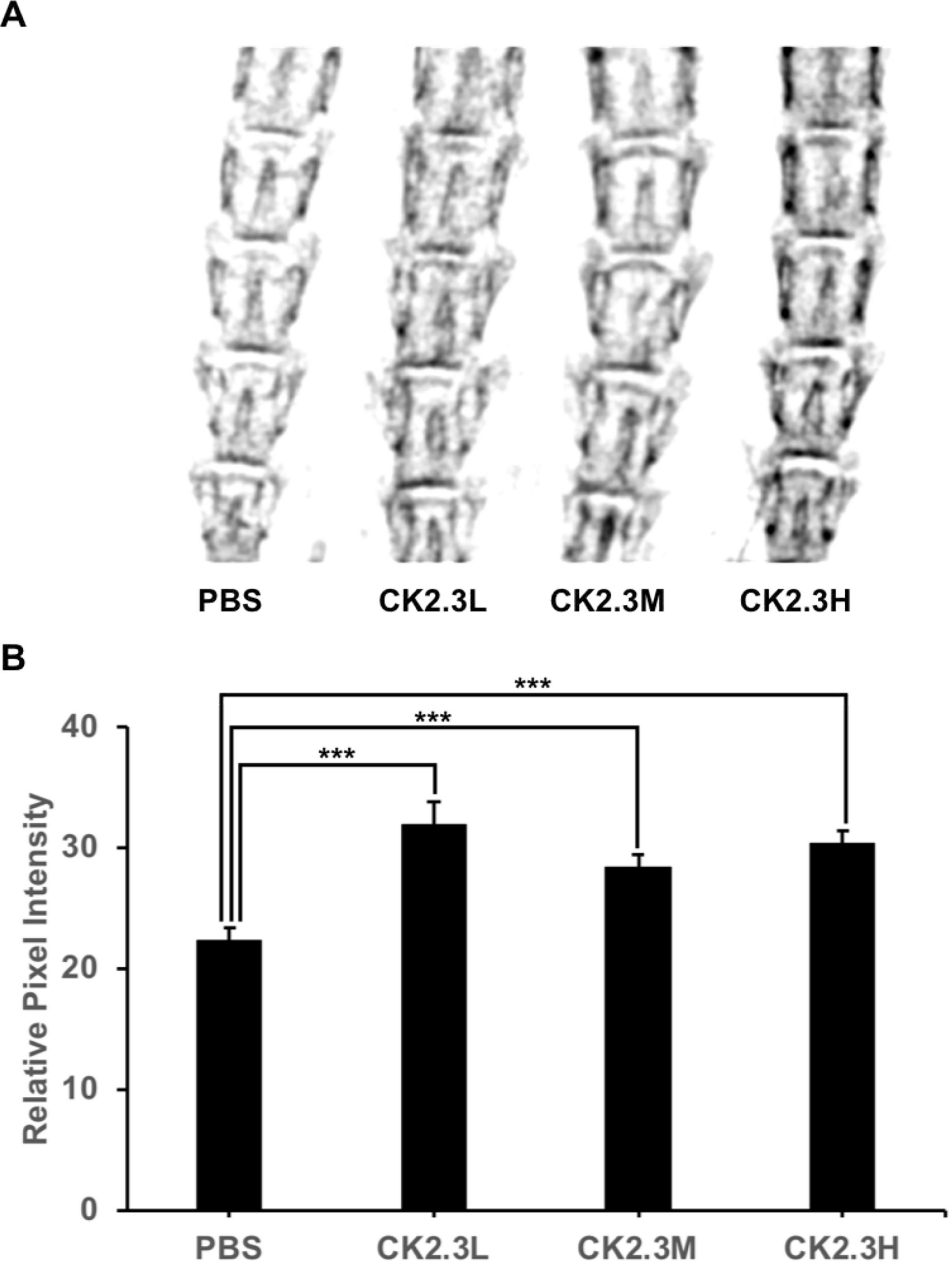
CK2.3 increased lumbar spine BMD. CK2.3 significantly enhanced lumbar spine BMD four weeks (p-value<0.0001) after the first injection. A) X-ray images of lumbar spine, B) Quantitative analysis of x-ray images. Six mice were used per group of treatment. Statistically significance was performed by ANOVA followed by Tukey-Kramer (***=significant, p-value<0.0001).

Furthermore, CK2.3 significantly enhanced lumbar spine BMD four weeks (p-value<0.0001) after the initial injection at CK2.3L (n=6, 31.98 ± 1.81), CK2.3M (n=6, 28.39 ± 1.08), and CK2.3H (n=6, 30.36 ± 1.11) concentration when compared to PBS control (n=6, 22.39 ± 1.03) (Figure 3).

**Figure 4:**
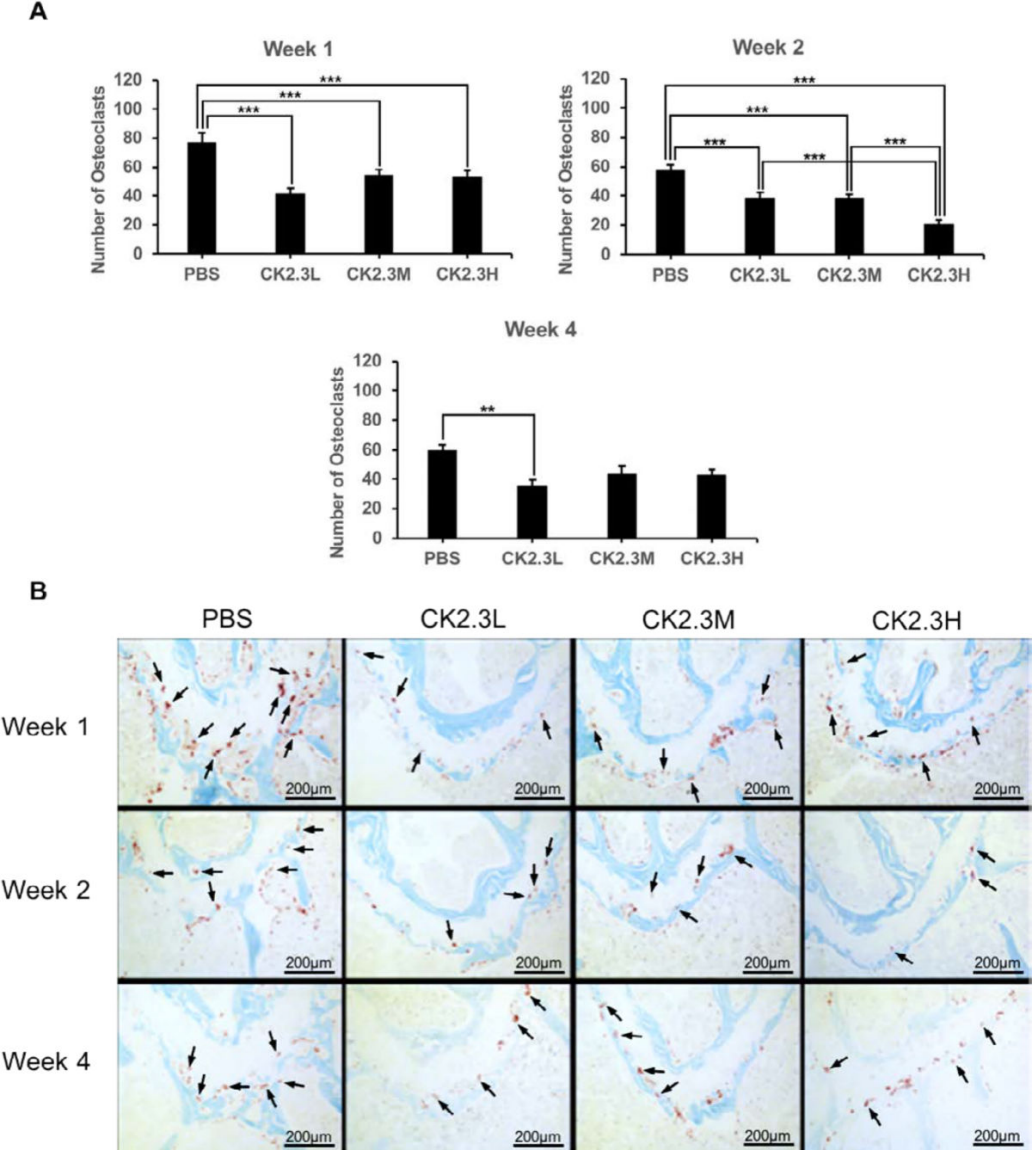
CK2.3 suppressed osteoclastogenesis *in vivo*. Images of distal growth plate region of femur were taken. Osteoclasts were stained with TRAP staining assay and appeared as purple. Osteoclast number was significantly decreased one week (p-value<0.0001) and two weeks (p-value<0.0001) after the first injection at all concentrations. But only CK2.3L suppressed osteoclastogenesis four weeks (p-value<0.01) after the first injection. A) quantify number of osteoclasts in each treatment at different time points. B) representative images of each treatment at different time points. Five to six mice were used per treatment group at each time point. Arrows point to representative osteoclasts (not all osteoclasts were pointed out). Statistically significance was performed by ANOVA followed by Tukey-Kramer (** and ***=significant, p-value<0.01 and p-value<0.0001, respectively).

Next, we examined the effect of CK2.3 dosage on osteoclast differentiation in the growth plate region of 6-month-old mice (Figure 4). Osteoclast was stained for TRAP, a marker of osteoclasts. Interestingly, our findings showed that osteoclast counts were significantly suppressed in the (distal) growth plate region at CK2.3L (n=6, 41.5 ± 4.0 and 38.7 ± 3.7), CK2.3M ((n=6)54.2 ± 4.1 and (n=5)38.7 ± 2.4), and CK2.3H (n=6, 52.6 ± 4.8 and 20.5 ± 2.8) when compare to PBS control (n=6, 76.7 ± 6.8 and 57.2 ± 4.0) one week (p-value<0.0001) and two weeks (p-value<0.0001), respectively, after the initial injection (Figure 4A). At week 4 (p-value=0.01), only CK2.3L (n=6, 35.9 ± 3.9) still exhibited inhibitory effect on osteoclastogenesis while CK2.3M (n=6, 43.8 ± 5.0) and CK2.3H (n=6, 42.9 ± 3.9) had no effect on osteoclastogenesis when compared to PBS control (n=6, 59.3 ± 4.3) (Figure 4A).

**Figure 5:**
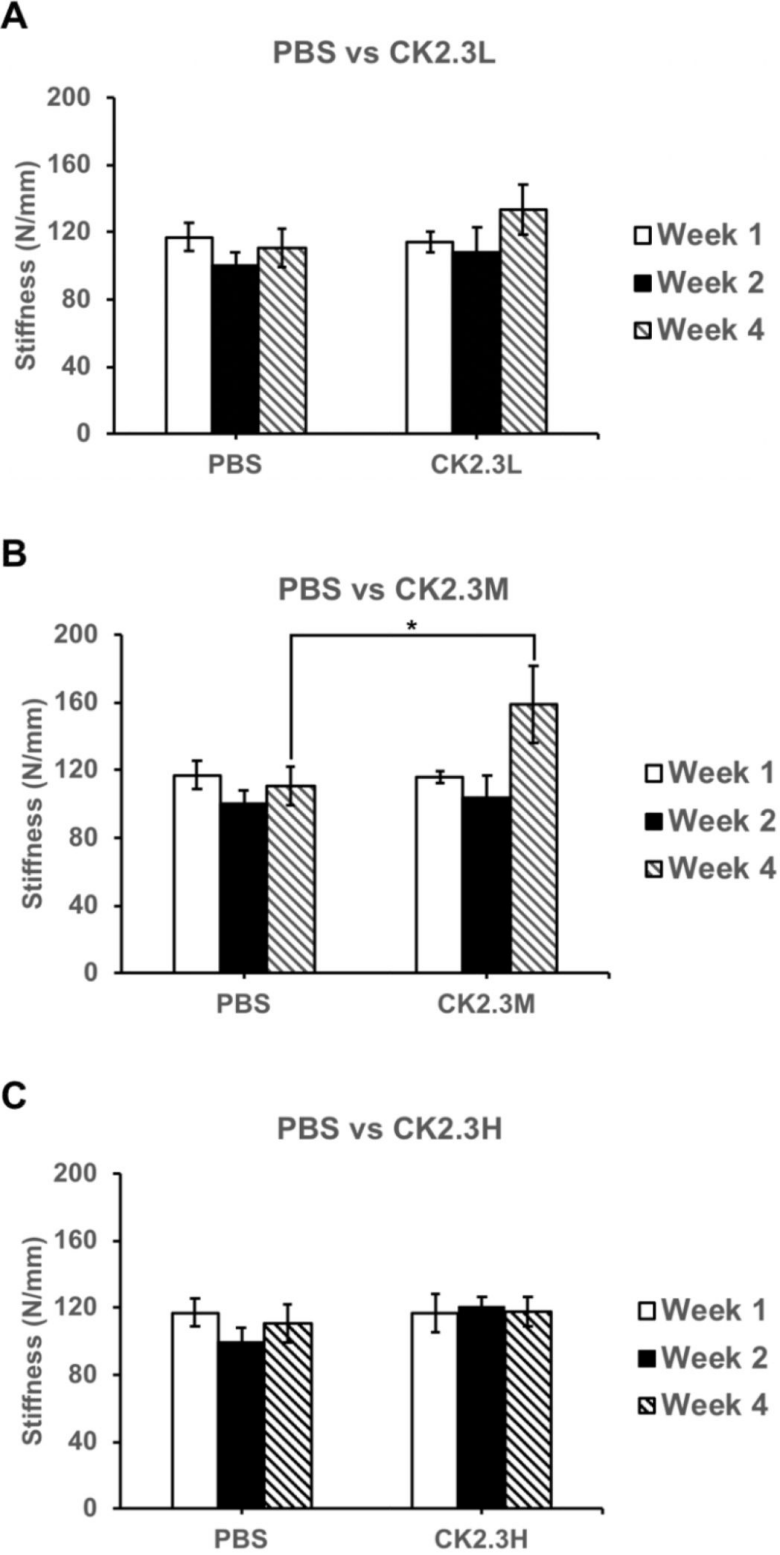
CK2.3 increased femoral shaft stiffness at medium concentration. CK2.3 didn’t affect femoral shaft stiffness at A) low or C) high concentration at all-time points. CK2.3 increased femoral shaft stiffness at B) medium concentration only four weeks after the initial injection (p-value<0.05). Five to six mice were used per group of treatment at each time point. Statistically significance was performed by ANOVA followed by Tukey-Kramer (*=significant, p-value<0.05).

Interestingly when investigating the effect of CK2.3 on the stiffness of the femoral shaft, we observed that CK2.3 significantly increased the stiffness of the femoral shaft by 43.6% (p-value<0.05) in mice injected with CK2.3M (n=6, 158.90 N/mm ± 23.17) only four weeks after the initial injection relative to PBS injected group (n=6, 110.62 N/mm ± 11.34) (Figure 5). CK2.3 didn’t have any effect in mice injected with CK2.3L and CK2.3H (Figure 5A & 5C, respectively).

**Figure 6:**
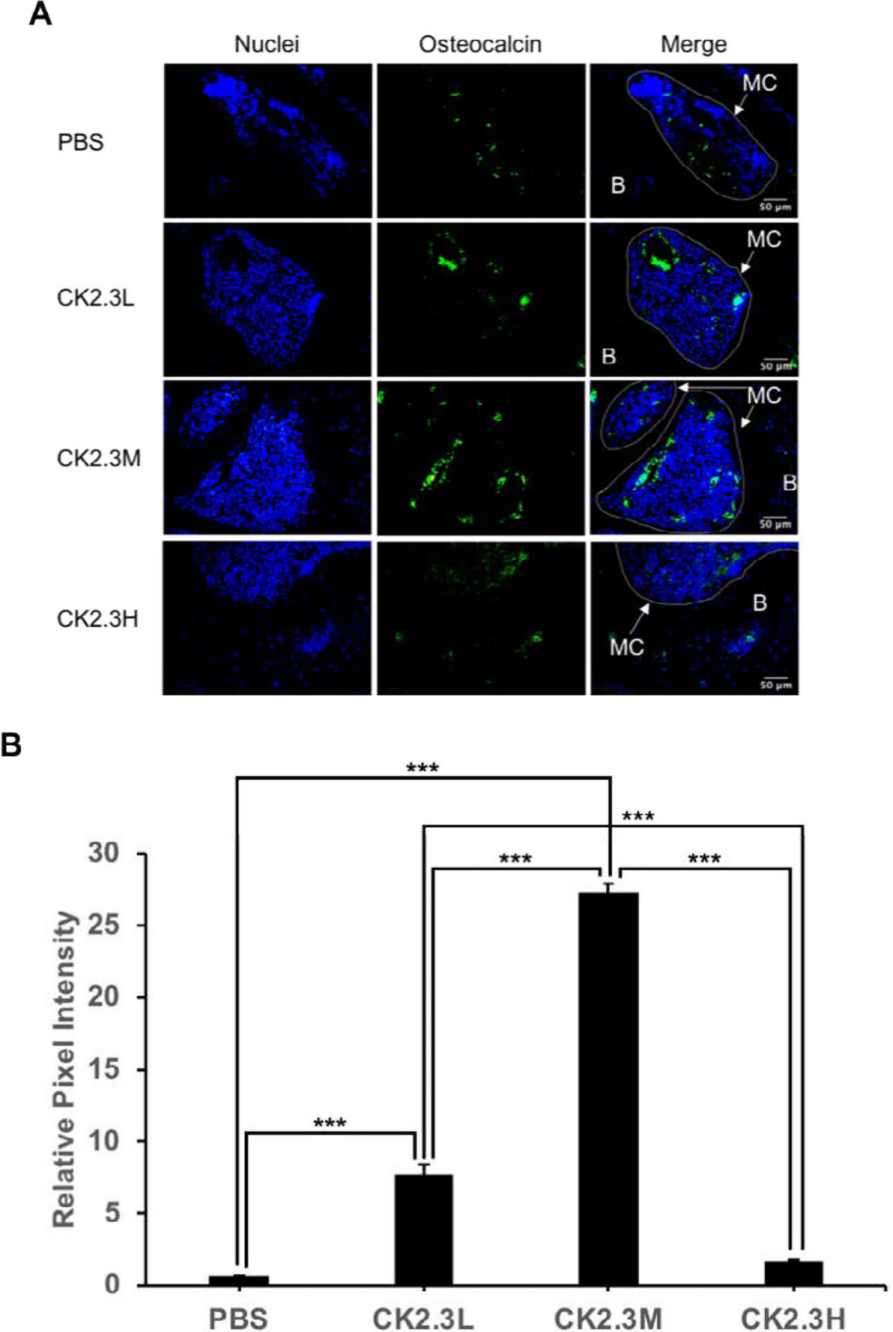
CK2.3 elevated osteocalcin expression in osteoblasts situated in femoral bone cavities. CK2.3 elevated osteocalcin expression at CK2.3L and CK2.3M four weeks after the first CK2.3 injection with CK2.3M was the most effective concentration. MC: marrow cavity; B: bone. Six mice were used per group of treatment. Statistically significance was performed by ANOVA followed by Tukey-Kramer (***=significant, p-value<0.0001).

**Figure 7:**
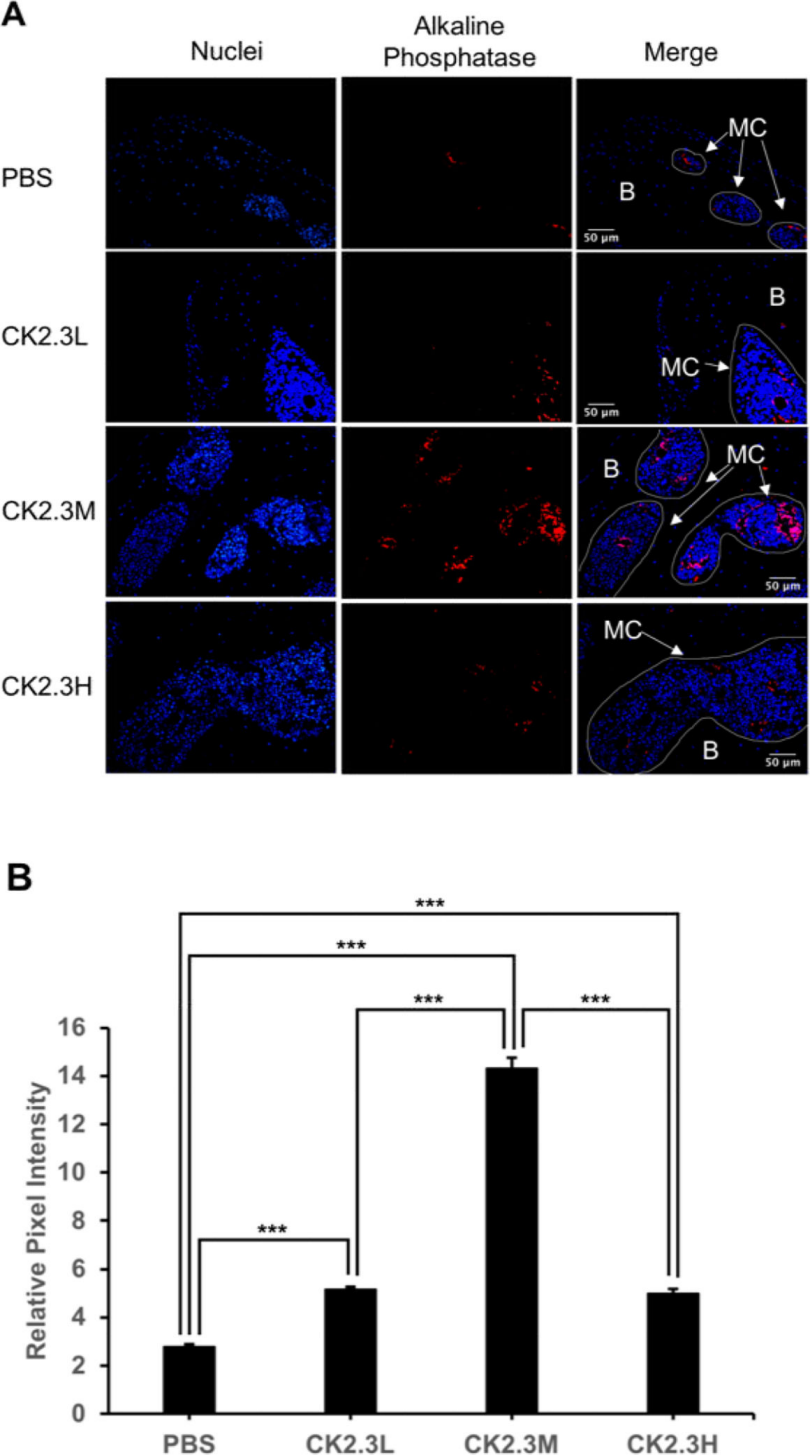
CK2.3 elevated alkaline phosphatase expression in osteoblasts situated in femoral bone cavities. CK2.3 elevated alkaline phosphatase expression at all concentrations four weeks after the first CK2.3 injection with CK2.3M was the most effective concentration. MC: marrow cavity; B: bone. Six mice were used per group of treatment. Statistically significance was performed by ANOVA followed by Tukey-Kramer (***=significant, p-value<0.0001).

To further investigate the effect of concentration of CK2.3 on osteoblastogenesis. We embedded right femurs in MMA, sectioned into thin slides (~5 μm), and labeled osteocalcin (green) and alkaline phosphatase (red), osteoblast markers. We observed a significant 12-fold and 44-old increase in osteocalcin expression at CK2.3L and CK2.3M, respectively, four weeks after the first CK2.3 injection (p-value<0.0001) (Figure 6). Similarly, we observed a significant 1.8-fold increase in alkaline phosphatase expression at both CK2.3L and C2.3H, and a peak 5.1-fold increase at CK2.3M four weeks after the first CK2.3 injection (p-value<0.0001) (Figure 7).

## Discussion

Osteoporosis is a bone disease that affects an estimated 10 million adults in the US [2]. Typically, treatments for osteoporosis include bisphosphonates and hormone therapy. However, these drugs often have detrimental side effects [4,5]. Other treatments, such as rhBMP2 therapy, focus on bone fracture healing by enhancing osteoblastogenesis [6]. Nevertheless, rhBMP2 also has limitations including its direct and indirect enhancement of osteoclastogenesis and bone resorption [8–11]. Our research led us to discover CK2 as a regulatory protein of BMP2 signaling pathway [13]. The blocking of the binding of CK2 at one of the three potential binding sites on BMPRIa resulted in enhancing bone mineralization in vitro and in vivo and suppressing osteoclastogenesis and bone resorption *in vitro* [14]. In this study, we used 6-month-old female mice. It was reported that female mice had lower total body BMD and bone volume to tissue volume ratio (BV/TV) than male mice at 4 to 20-month-old [25]. In addition, their study reported that both male and female mice were reported to have a decrease in total bone BMD and BV/TV. Furthermore, it shows that women over the age of 50 have 4 times higher rate of osteoporosis and 2 times higher rate of osteopenia than men [26]. Thus, for this study female mice were chosen.

In this study for the first time, we showed the effect of CK2.3 on osteoclastogenesis and bone mineral density in the femur in vivo and the lumbar spine in vivo, respectively, at different concentrations of dosage. The dosage was based on a previous publication where female C57BL/6J mice were injected with CK2.3 (2.3μg/kg per mouse) [14]. Here, we wanted to study the effect of low (three times lower than previously injected dosage) and high (three times higher than previously injected dosage) concentrations of CK2.3. The effect of CK2.3 on trabecular BMD and lumbar spine BMD was observed after four weeks post initial injection at all three dosages (Figures 1–3). We also observed an increasing trend of BMD in response to an increase in CK2.3 concentration of injection. This observation was supported by the analyses of MAR (Figure 2).

An interesting finding of this study was the enhancement of femoral shaft stiffness which was only observed at CK2.3 medium concentration (2.3 μg/kg) four weeks after the first CK2.3 injection (Figure 5). Bone structural integrity is maintained via the process of bone remodeling [27]. Thus, disruption of bone remodeling process can lead to a weak structural integrity of bone. It was reported that strong inhibitors of osteoclast activity such as bisphosphonates weakened the structural integrity of bone and increased formation of micro-fractures [28]. Examining the osteoclastogenesis of the growth plate region of the femurs, we observed that the inhibitory effect of CK2.3 lasted up to four weeks at CK2.3 low concentration (0.76 μg/ kg per mouse) (Figure 4A). On the other hand, the inhibitory effect of CK2.3 increased as treatment was continued up two weeks at CK2.3 high concentration (6.9 μg/kg) and lost its effect by week 4 of treatment (Figure 4A). Only at CK2.3 medium concentration, the inhibitory effect of CK2.3 was maintained at a same level until losing its effect at week 4 of treatment (Figure 4A). Investigating osteoblast formation, it showed that at CK2.3 medium concentration, expression of osteocalcin and alkaline phosphatase, markers of osteoblast, extremely increased at week 4 of treatment (Figure 6). These findings suggested while acting as a moderate inhibitor of osteoclast activity with short half-life and as a strong promoter of osteoblastogenesis at CK2.3 medium concentration, CK2.3 maintained the structural integrity of bone.

In this study, we found the lack of dose-dependent response in trabecular BMD (Figure 1) and lumbar spine BMD (Figure 3). On the other hand, dose-dependent response was observed in mineral apposition rate (Figure 2), osteoclastogenesis (Figure 4), femur stiffness (Figure 5), and osteocalcin expression (Figure 6). The lack of dosedependent response in BMD might due to the observation that higher concentration of CK2.3 caused more bone formation (Figure 2). On the other hand, less osteoclast formation was found at higher concentration of CK2.3 (Figure 4). Thus, this might have balanced out the BMD as the result of bone remodeling process as a whole.

Studies that were done on current treatments of osteoporosis such at BMP2 and PTH showed that long-term usage of these compounds resulted in enhancing osteoclast differentiation and bone resorption [7,29,30]. It could be speculated that long term treatment of osteoporosis using BMP2 and PTH might increase osteoclast differentiation and bone resorption due to the direct and indirect effects (via osteoblasts) on osteoclasts. However, due to the unique characteristic of CK2.3 that is, enhancing osteoblastogenesis while suppressing osteoclastogenesis, long term treatment using CK2.3, thus, does not increase osteoclast differentiation (Figure 4). The positive regulation of osteoclastogenesis by CK2 was reported [31,32]. Even though it is still unknown to what extent CK2 activity is inhibited by the binding of CK2.3, it is possible that CK2.3 suppressed osteoclast differentiation via inhibition of CK2 activity.

It shows that male sex hormone, testosterone, also has similar effect on mediating osteoclastogenesis and osteoblastogenesis as female sex hormone, estrogen [33]. Therefore, future study on male subject should be performed to determine any gender disparities in bone formation resulted by the effect of CK2.3.

## Conclusions

This study elucidated the important of the effect of CK2.3 concentration on bone biology. At the right concentration, CK2.3 showed to be an excellent bone promoter while maintaining the structural integrity of the bone. CK2.3 has tremendous potential as a novel therapeutic treatment for osteoporosis.

## Abbreviations

PTH: Parathyroid Hormone
PTHrP: Parathyroid Hormone Related-Protein
hPTHrP: Human Parathyroid Hormone Related-Protein
ALP: Alkaline Phosphatase
TRAC-5B: Tartrate-resistant Acid Phosphatase Form 5b
BMP2: Bone Morphogenetic Protein 2
CK2: Casein Kinase 2
BMPRIa: Bone Morphogenetic Protein Receptor Type Ia
BMD: Bone Mineral Density
SPA: Single Photon Absorptionmetry
MMA: Methyl Methacrylate
TRAP: Tartrate-resistant Acid Phosphatase
MAR: Mineral Apposition Rate
